# Supplementary material for: COVID-19 vaccination beliefs, attitudes, and behaviours among health and social care workers in the UK: A mixed-methods study
Source: PLoS One. 2022 Jan 24;17(1):e0260949. doi: 10.1371/journal.pone.0260949 (PMC8786153; doi:10.1371/journal.pone.0260949)
Supplement: S2 Appendix — (DOCX) [file pone.0260949.s002.docx]

# Supporting Information 2 – Recoding of variables

## Combining categorical variables for analysis

### Ethnicity

The following outlines how each of the Ethnicity subcategories, used in the statistical analysis, were constructed from the original survey question. **(1)** **White British and White Irish** is a combination of the White British (n=1051) and White Irish (n=51) categories. **(2) White Other** is a combination of Gypsy or Irish Traveller(n=1) and Other White Backgrounds (n=93). **(3)** **Black or Black British African or Mixed Black African** is a combination of the Black and Black British African (n=155) and the Mixed White and Black African (n=13) categories. **(4) Black or Black British Caribbean or Mixed Black Caribbean** is a combination of the Black or Black British Caribbean (n=51) and the Mixed White and Black Caribbean (n=15) categories. **(5) Asian or Asian British Indian** was a single answer in the original questions (n=264). **(6) Other South East Asian or Mixed Asian** is a combination of Asian or Asian British Pakistani (n=32), Asian or Asian British Bangladeshi (n=11), Asian or Asian British Other Asian Backgrounds (n=50), and the Mixed White and Asian (n=16) category. **(7) Other Ethnic Minorities** is a combination of Arab (n=8), Chinese (n=14), Black or Black British any other Black Background (n=15), Mixed other mixed background (n=19) and Other ethnic minority not represented by these options (n=34).

### Job role

The following outlines how each of the Job role subcategories, used in the statistical analysis, were constructed from the original survey question. **(1) Allied Health Professionals** was a single answer in the original questions **(2) Medical** was a single answer in the original questions **(3) Registered Nursing and Midwives** was a single answer in the original questions **(4) Nursing or Healthcare** assistant was a single answer in the original questions **(5) Social Care** all participants that indicated that their sector was social care as there was not enough data to delimitate further **(6) Other** is a combination of Ambulance (operational), Public Health, Commissioning, Wider Healthcare Team and General Management.

## Combining continuous variables

### COVID-19 vaccine attitudes and beliefs

To reduce the number of attitude and belief variables included in the COVID-19 vaccine uptake regression analysis we conducted a factor analysis to determine which items were suitable to combine into a single variable. Below and Table 1 outline the results of a factor analysis that we conducted on these variables.

Component 1: Eigenvalue = 5.453, Variance = 45.4

Component 2: Eigenvalue = 1.308, Variance = 10.9

Component 3: Eigenvalue = 1.201, Variance = 10.0

### Table 1: Pattern Matrix for the three components identified in factor analysis (principle components analysis)

| **Statement** | **Component 1** | **Component 2** | **Component 3** |
| --- | --- | --- | --- |
| *“I think COVID-19 is deadlier than seasonal flu”* | 0.534 | 0.377 | 0.170 |
| *“I think it's important for social/health care workers to get a COVID-19 vaccine to protect themselves”* | 0.878 | 0.312 | -0.001 |
| *“I think it’s important for social/health care workers to get a COVID-19 vaccine to protect their families”* | 0.859 | 0.365 | -0.007 |
| *“I think it’s important for social/health care workers to get a COVID-19 vaccine to protect their patients”* | 0.838 | 0.329 | 0.020 |
| *“I think that COVID-19 vaccines are safe”* | 0.802 | -0.207 | -0.139 |
| *“I think that COVID-19 vaccines are effective”* | 0.764 | -0.175 | -0.116 |
| *“I think it is important for people to get vaccinated against COVID-19 to get life back to 'normal'”* | 0.773 | 0.137 | -0.017 |
| *“I feel well informed about COVID-19 vaccination”* | 0.597 | -0.385 | -0.135 |
| *“My family and friends expect me to accept a COVID-19 vaccine”* | 0.559 | -0.330 | 0.490 |
| *“My colleagues expect me to accept a COVID-19 vaccine”* | 0.372 | -0.406 | 0.694 |
| *“I felt under pressure from my employer to get a COVID-19 vaccine”* | -0.352 | 0.195 | 0.539 |
| *“I am worried about getting side-effects from a COVID-19 vaccine”* | -0.450 | 0.524 | 0.328 |

After discussion with the co-authors it was decided that the following items could be combined into a single variable: **(1)** *“I think it's important for social/health care workers to get a COVID-19 vaccine to protect themselves”* **(2)** *“I think it’s important for social/health care workers to get a COVID-19 vaccine to protect their families”* **(3)** *“I think it’s important for social/health care workers to get a COVID-19 vaccine to protect their patients”* **(4)** *“I think that COVID-19 vaccines are safe”* **(5)** *“I think that COVID-19 vaccines are effective”* **(6)** *“I think it is important for people to get vaccinated against COVID-19 to get life back to 'normal'”.* This new variable was assigned the label: **Combined COVID-19 vaccine beliefs (important, safe, and effective).** The Cronbach’s Alpha for these variables was .918 suggesting high internal consistency.

Similarly, the items *“My family and friends expect me to accept a COVID-19 vaccine”* and *“My colleagues expect me to accept a COVID-19 vaccine”* were combined to form a variable we labelled **Social norms to vaccinate against COVID-19.** The Cronbach’s Alpha for these variables was .661 suggesting fair to good internal consistency. Initially we included *“I felt under pressure from my employer to get a COVID-19 vaccine”* in this combined variable but later separated it and used it as a single item variable due to the importance participants placed on pressure within qualitative interviews.

*“I think COVID-19 is deadlier than seasonal flu”*, *“I feel well informed about COVID-19 vaccination”*, and *“I am worried about getting side-effects from a COVID-19 vaccine”* were kept as single item variables in the analysis due to them not clearly loading on to any of the components suggested by the factor analysis.

### Trust in sources of information

To reduce the number of trust variables included in the COVID-19 vaccine uptake regression analysis we conducted a factor analysis to determine which items were suitable to combine into a single variable. Below and Table 2 outline the results of a factor analysis that we conducted on these variables.

Component 1: Eigenvalue = 4.768, Variance = 39.7

Component 2: Eigenvalue = 2.07, Variance = 17.2

Component 3: Eigenvalue = 1.033, Variance = 8.6

Table 2: Pattern Matrix for the three components identified in factor analysis (principle components analysis)

| **I trust the advice on Covid-19 vaccination given by…** | **Component 1** | **Component 2** | **Component 3** |
| --- | --- | --- | --- |
| *My work colleagues* | 0.313 | 0.060 | -0.482 |
| *Social media* | -0.218 | 0.734 | -0.111 |
| *Community leaders* | 0.149 | 0.679 | -0.120 |
| *Religious leaders* | -0.072 | 0.731 | -0.129 |
| *NHS* | 0.842 | 0.078 | -0.004 |
| *News media (e.g. print or online newspapers, radio, and television news broadcasts)* | 0.243 | 0.636 | 0.030 |
| *Government* | 0.571 | 0.438 | 0.168 |
| *Family* | -0.012 | 0.130 | -0.833 |
| *Friends* | -0.023 | 0.080 | -0.889 |
| *Scientists involved in COVID-19 vaccine development* | 0.828 | -0.117 | -0.096 |
| *Public Health England* | 0.852 | 0.072 | 0.088 |
| *Health Professionals* | 0.816 | -0.124 | -0.233 |

After decision with co-authors the following combination of items were suggested for use in the COVID-19 vaccine uptake regression analysis.

**Trust in health system sources**. Containing: *NHS*, *Scientists involved in COVID-19 vaccine development*, *Public Health England*, and *Health Professionals*. The Cronbach’s Alpha for these variables was .876 suggesting high internal consistency.

**Trust in non-health system sources**. Containing: *Social Media*, *Community leaders*, *Religious leaders,* and *News media.* The Cronbach’s Alpha for these variables was .738 suggesting high internal consistency.

**Trust in Friends and Family members**. Containing: *Family* and *Friends*. The Cronbach’s Alpha for these variables was .876 suggesting high internal consistency.

The following two items did not clearly load onto any of the three components suggested by the factor analysis: **(1)** *My work colleagues*, and **(2)** *Government*. As such we included these as single item variables within our analysis.
